# Undiagnosed Acute Viral Febrile Illnesses, Sierra Leone

**DOI:** 10.3201/eid2007.131265

**Published:** 2014-07

**Authors:** Randal J. Schoepp, Cynthia A. Rossi, Sheik H. Khan, Augustine Goba, Joseph N. Fair

**Affiliations:** US Army Medical Research Institute of Infectious Diseases, Fort Detrick, Maryland, USA (R.J. Schoepp, C.A. Rossi);; Kenema Government Hospital, Kenema, Sierra Leone (S.H. Khan, A. Goba);; Metabiota, San Francisco, California, USA (J.N. Fair)

**Keywords:** Sierra Leone, undiagnosed, febrile, viruses, arthropod-borne, hemorrhagic fever, diagnostics, serologic, immunodiagnostics, ELISA, IgM, West Africa, Lassa virus, vector-borne infections

## Abstract

Sierra Leone in West Africa is in a Lassa fever–hyperendemic region that also includes Guinea and Liberia. Each year, suspected Lassa fever cases result in submission of ≈500–700 samples to the Kenema Government Hospital Lassa Diagnostic Laboratory in eastern Sierra Leone. Generally only 30%–40% of samples tested are positive for Lassa virus (LASV) antigen and/or LASV-specific IgM; thus, 60%–70% of these patients have acute diseases of unknown origin. To investigate what other arthropod-borne and hemorrhagic fever viral diseases might cause serious illness in this region and mimic Lassa fever, we tested patient serum samples that were negative for malaria parasites and LASV. Using IgM-capture ELISAs, we evaluated samples for antibodies to arthropod-borne and other hemorrhagic fever viruses. Approximately 25% of LASV-negative patients had IgM to dengue, West Nile, yellow fever, Rift Valley fever, chikungunya, Ebola, and Marburg viruses but not to Crimean-Congo hemorrhagic fever virus.

## Abstract 2

Various arthropod-borne and hemorrhagic fever viruses should be considered when Lassa fever is suspected.

The West African country of Sierra Leone is located in a Lassa fever–hyperendemic region that also includes Guinea and Liberia. The causative agent of Lassa fever is Lassa virus (LASV), a member of the *Arenaviridae* family. Lassa fever is a severe, often fatal, hemorrhagic illness; the virus causes 100,000–300,000 infections and 5,000 deaths each year in the region (*1*).

In 2002, Sierra Leone emerged from a brutal 11-year civil war that left the country with little infrastructure and much of its formal economy destroyed. Today Sierra Leone is undergoing substantial economic growth; however, poverty, unemployment, and inadequate health care remain major challenges. Through the Mano River Union–Lassa Fever Network, a variety of organizations are building diagnostic capacity for Lassa fever that will lead to better understanding of the disease and its treatment (*2*–*5*). These scientific efforts are centered in eastern Sierra Leone at the Kenema Government Hospital (Kenema, Sierra Leone) within the Lassa fever–hyperendemic region (Figure). The Lassa Fever Ward, a 16-bed facility on the hospital grounds, is dedicated to treating patients suspected of having Lassa fever and is supported by the Lassa Diagnostic Laboratory.

**Figure:**
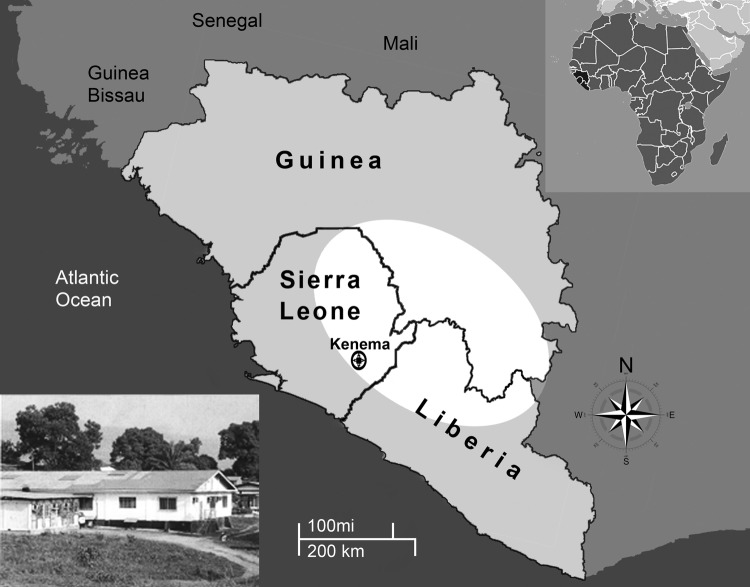
Lassa fever–hyperendemic region (white area) comprising parts of Guinea, Sierra Leone, and Liberia in West Africa. Insert image: Lassa Diagnostic Laboratory, Kenema Government Hospital, Kenema, Sierra Leone.

Each year, suspected Lassa fever infections result in submission of ≈500–700 samples to the Kenema Government Hospital Lassa Diagnostic Laboratory (J. Bangura, unpub. data). Samples come from throughout the Lassa fever–hyperendemic region and initially are screened for malaria by thick blood smear and, if negative, are tested for LASV. LASV infection is determined by the presence of virus detected by an antigen-detection ELISA and by the presence of IgM determined by using an IgM-capture ELISA. Generally only 30%–40% of samples tested are positive for LASV antigen and/or LASV-specific IgM; therefore, 60%–70% of patients have acute diseases of unknown origin. We investigated what other arthropod-borne and hemorrhagic fever viral diseases might be causing serious illness in the region and confounding the diagnosis of Lassa fever. We tested samples from these patients using IgM-capture ELISAs to virus pathogens that could occur in the region and mimic Lassa fever. We tested for IgM to dengue virus (DENV), West Nile virus (WNV), yellow fever virus (YFV), Rift Valley fever virus (RVFV), chikungunya virus (CHIKV), Ebola virus (EBOV), Marburg virus (MBGV), and Crimean-Congo hemorrhagic fever virus (CCHFV). Follow up analyses included IgG ELISAs and/or confirmatory plaque-reduction neutralization tests (PRNTs). This study provides a better understanding of the differential diagnoses for Lassa fever in the region, which can lead to improved diagnostic capability and disease treatment.

## Materials and Methods

### Patient Samples

Blood samples collected during October 2006–October 2008 from patients with suspected Lassa fever were submitted to the Lassa Diagnostic Laboratory. Most samples were from acutely ill patients from eastern Sierra Leone; some were submitted from Liberia and a few from Guinea. Samples were from patients whose illness met a surveillance case definition for Lassa fever (Table 1) and were processed as described elsewhere (*5*). Samples that were malaria negative by thick blood smear and were from patients whose illness failed to respond to antimicrobial drugs were initially tested in the Lassa Diagnostic Laboratory for LASV antigen by antigen detection ELISA and for LASV-specific IgM by IgM-capture ELISA. LASV-negative samples were then tested for IgM to DENV, WNV, YFV, RVFV, and CHIKV before being transferred to the US Army Medical Research Institute of Infectious Diseases for additional testing for IgM to EBOV, MBGV, and CCHFV. IgM-positive samples were tested for IgG by ELISA and/or confirmatory PRNT, depending on the volume of sample available.

**Table 1:** Case definition used to detect suspected Lassa fever at Kenema Government Hospital, Kenema, Sierra Leone, October 2006–October 2008*

Major criteria	Minor criteria
Known exposure to person with suspected Lassa fever	General myalgia or arthralgia
Abnormal bleeding (from mouth, nose, rectum, or vagina)	Headache
Edema of the neck and/or face	Sore throat
Conjunctivitis or subconjunctival hemorrhage	Vomiting
Spontaneous abortion	Abdominal pain/tenderness
Petechial or hemorrhagic rash	Retrosternal pain
Onset of tinnitus or altered hearing	Cough
Persistent hypotension	Diarrhea
Elevated liver transaminases (aspartate aminotransferase higher than alanine aminotransferase)	Profuse weakness Proteinuria Leukopenia (leukocytes <4,000 μL)

*To be tested for suspected Lassa fever, a patient had to have a fever >38°C and not respond to appropriate antimalarial and antimicrobial drug treatment within 72 h.

Research on human subjects was conducted in compliance with US Department of Defense, federal, and state statutes and regulations relating to the protection of human subjects, and adhered to principles identified in the Belmont Report (1979) (www.hhs.gov/ohrp/humansubjects/guidance/belmont.html). All data were gathered and human subjects research was conducted under an institutional review board–approved protocol (no. HP-09-32).

### Antigens and Antiserum

Viruses used in production of ELISA antigenic materials were the 4 DENVs (DENV-1 Hawaii, DENV-2 New Guinea C strain [*6*,*7*], DENV-3 H87 strain, and DENV-4 H241 strain [*8*]); WNV EG101 strain (*9*); YFV Asibi strain (IgM ELISA) or YFV 17D strain (Connaught) (IgG ELISA) (*10*); CHIKV Indo23574 strain; RVFV ZH 501 strain (*11*); EBOV Zaire-76 strain (*12*); MBGV Musoke strain (*13*); CCHFV IbAr10200 strain (*14*); and LASV Josiah strain (*15*). The antigens were prepared and optimized as previously described (*16*). All viruses were propagated at Biosafety Level 3 or 4, as appropriate. Briefly, the viruses were grown in appropriate continuous cell lines until cytopathic effects were observed in 50%–75% of the cells. Cell culture supernatants, used in the IgM- and IgG-capture ELISAs, were clarified by centrifugation, inactivated by treatment with 0.3% β-propiolactone, aliquoted, and stored at −70°C. Cell lysates for the IgG sandwich ELISAs were produced from virus-infected cell pellets that were resuspended, sonicated, clarified, aliquoted, and stored at −70°C. Virus-infected cell culture supernatants and cell lysates were inactivated by γ-irradiation (3 × 10^6^ rad) and safety tested to ensure inactivation. Optimal dilutions of antigens were determined by checkerboard titrations against virus-specific antibodies. Negative or mock antigens, both supernatant and cell lysate, used as negative controls, were prepared from uninfected cell monolayers as described above.

### IgM-Capture ELISA

The IgM-capture ELISAs were performed as described elsewhere (*17*,*18*). Briefly, 96-well round bottom polyvinyl chloride microtiter plates were coated with diluted goat anti–human IgM heavy chain capture antibody overnight at 4°C. The capture plates were washed, and then patient samples, diluted 1:100, were added and incubated for 1 h at 37°C. With every assay, we included at least 1 known positive control serum sample to ensure assay was working. and 4 known negative control serum samples were used to determine assay cutoff. After washing the plates, the inactivated cell culture supernatant antigen or mock antigen was added, and the plates were incubated for 1 h at 37°C. Unlike the other assays that used a single virus strain, the DENV IgM ELISA antigen was a mixture of all 4 viruses and was recognized by antibodies against each (*19*). Samples were tested in duplicate against each virus and mock antigen. After the plates were washed, a secondary detector antibody (mouse or rabbit polyclonal serum antibody titers raised against the target virus) was added, and the plates were incubated for 1 h at 37°C. After additional washing, horseradish peroxidase (HRP)–labeled antidetector species antibody (goat anti–mouse IgG, heavy and light chain, or HRP-labeled goat anti–rabbit IgG, heavy and light chain), was added to the mixture, and the plates were incubated for 1 h at 37°C. The plates were washed again, ABTS (2, 2'-azino-di[3-ethylbenzthiazoline-6-sulfonate]) substrate was added, and the plates were incubated for 30 min at 37°C. The optical densities (OD) were determined at 410 nm in an automated ELISA reader. We determined an adjusted OD for each sample by subtracting the average mock antigen OD from the average positive antigen OD. For each assay, the mean deviation and SD of the adjusted ODs were determined for all 4 negative control samples. The cutoff of each assay was the mean OD plus 3 SDs rounded up to the nearest tenth. This OD was typically 0.2. A sample was considered positive if the OD was greater than or equal to this OD cutoff.

### IgG ELISA

We conducted the IgG ELISAs on selected serum samples using a modification of the IgM-capture ELISAs described above. Briefly, polyvinyl chloride microtiter plates were coated with inactivated cell lysate or mock antigen overnight at 4°C (*19*). To detect RVFV IgG, we used a sandwich IgG ELISA; plates were coated with an RVFV-specific nucleocapsid monoclonal antibody overnight at 4°C to capture inactivated cell culture supernatant onto the plate surface (1 h at 37°C) (*18*). Antigen-coated plates were washed; diluted patient samples (1:100) were added, and plates were incubated for 1 h at 37°C. Samples were tested in duplicate against each virus and mock antigen, and we included at least 1 known positive control sample and 4 known negative control samples with every assay. After the plates were washed, diluted HRP-labeled mouse anti–human IgG (Fc-specific) conjugate was added, and the plates were incubated for 1 h at 37°C. After again washing the plates, ABTS substrate was added, and the plates were incubated for 30 min at 37°C, and the absorbance at 410 nm was determined. Mathematical calculations and assay cutoffs were determined as described for the IgM-capture ELISA.

### PRNT

PRNTs were conducted on selected serum samples as described elsewhere (*20*). Briefly, heat-inactivated serum samples were diluted 4-fold from 1:10 to 1:10,240 and were tested for their ability to neutralize ≈100 PFU of the challenge virus. Each sample dilution was tested in duplicate. Both known positive and negative control serum samples were included with every assay. Serum–virus mixtures were incubated overnight at 4°C and then inoculated onto 85%–100% confluent monolayers of the appropriate cell lines grown in 6-well tissue culture plates. After incubation for 1 h at 37°C, a nutrient 0.5%–1% agarose overlay was added, and plates were incubated at 37°C for the appropriate number of days for the virus, then stained with a second overlay containing 4%–5% neutral red; plaques were counted 24–48 h later. To detect a wider range of viruses neutralized by the serum, we recorded the reciprocal of the highest serum dilution reducing 50% of the plaque assay dose. A titer >10 was considered positive. The virus strains used in the PRNT were the same as the strains used for ELISA antigen, with the exception of WNV(NY99 strain) (*21*). In addition, the PRNT used the alphaviruses, o’nyong-nyong (ONNV), Semliki Forest (SFV), and Sindbis (SINV); and the ebolaviruses, Sudan Gulu strain (SUDV) and Taï Forest (Cȏte d’Ivoire) virus (TAFV).

## Results

We tested serum samples from 253 patients submitted to the Lassa Diagnostic Laboratory during 2006–2008 for IgM to the arthropod-borne and hemorrhagic fever viruses of interest (Table 2). Because of limited amounts of serum, not all samples were tested for antibodies to all viruses. Of the arthropod-borne viruses, the prevalence of DENV antibodies (4.3%) was highest, followed by CHIKV (4.0%). The prevalences of other viruses were <3.0%; WNV, 2.8%; YFV, 2.5%; and RVFV, 2.0% of patients tested. No antibodies to the tick-borne virus, CCHFV, were found in any samples tested. Antibody prevalence to the hemorrhagic fever viruses, EBOV and MBGV, were 8.6% and 3.6%, respectively.

**Table 2:** Patients’ antibody reactions to arthropod-borne and hemorrhagic fever virus antigens, Lassa Diagnostic Laboratory, Kenema, Sierra Leone, October 2006–October 2008*

Virus	No. positive /total (%)	No. IgM only positive/total (%)
Dengue	11/253 (4.3)	6/250 (2.4)
West Nile	7/253 (2.8)	3/250 (1.2)
Yellow fever	5/201 (2.5)	5/201 (2.5)
Rift Valley fever	5/253 (2.0)	5/253 (2.0)
Chikungunya	10/253 (4.0)	5/253 (2.0)
Ebola	19/220 (8.6)	18/219 (8.2)
Marburg	8/220 (3.6)	7/219 (3.2)
Crimean-Congo hemorrhagic fever	0/220	Not tested
Total	65/253 (25.7)	49/253 (19.4)

*Detected by IgM-capture ELISA in serum samples submitted to Lassa Diagnostic Laboratory (Kenema, Sierra Leon) for suspected Lassa fever. All samples tested were malaria negative by thick blood smear and Lassa virus negative by antigen detection and IgM-capture ELISA when initially tested. Samples with sufficient volumes were tested for the presence of IgG to determine samples that were IgM positive only.

Samples positive for IgM and with sufficient volumes were tested for IgG. Of the 11 DENV IgM–positive patients, 6 (2.4% of total) were IgM positive only (Table 2). Of the 7 WNV IgM–positive patients, 3 (1.2%) were IgM positive only. Of the 10 CHIKV IgM–positive patients, 5 (2.0%) were IgM positive only. Of the 8 MBGV IgM–positive patients, 7 (3.2%) were IgM positive only. No IgG was detected in the patients identified as IgM positive for YFV, RVF, or EBOV. Two patients were IgM positive for both EBOV and MBGV; 1 sample neutralized EBOV in PRNT and 1 did not. MBGV PRNTs were not possible because of a lack of positive control serum samples. No IgM-only samples reacted in >1 flavivirus assay. Testing of patients with undiagnosed acute febrile illness for antibodies against the 8 viruses suggested a possible cause for illness in 25.7% of the patients originally suspected of having Lassa fever; of these, 19.4% demonstrated only an IgM response, suggesting an acute infection.

Malaria parasites and LASV are known to occur in the region, and we excluded samples with evidence of either because of our interest in undiagnosed acute febrile illnesses. However, the sample group was tested for LASV IgG to better understand the prevalence of Lassa fever. Of 237 patients, 25.5% were positive for LASV-specific IgG (data not shown). Because of the high number of IgG-positive patients, we retested the samples for LASV IgM and demonstrated that 7 (3.0%) of the patients tested had LASV-specific IgM.

Antibodies detected by ELISA cross-react, especially within a genus and particularly for antibodies elicited by alphaviruses and flaviviruses. Immunodiagnosis conventionally is confirmed by virus isolation or a rise in PRNT titer (*22*). We did not attempt to isolate viruses because the samples were heat inactivated to protect the laboratory personnel. Confirmation by PRNT ideally uses paired serum samples (acute- and convalescent- phase), demonstrating a 4-fold rise in titer. In our retrospective study, we had only acute-phase serum samples, but we performed PRNTs in an attempt to clarify the specific viruses causing severe disease in this region. Flavivirus-reactive patient serum, positive for IgM only, was tested for its ability to neutralize DENV-3, WNV, and YFV. Comparison of neutralizing titers could not attribute a specific virus as the cause of disease, with the exception of YFV (data not shown). Three of the 5 YFV IgM–positive serum samples demonstrated neutralizing titers to YFV and not to the other flaviviruses tested. These serum samples also were not reactive by IgM ELISA to any of the other 8 viruses tested. CHIKV-reactive patient serum, positive for IgM only, was tested for its ability to neutralize CHIKV, ONNV, SFV, and SINV (Table 3). Four of the 5 samples tested neutralized ONNV to a greater degree than CHIKV. We found no correlation between OD and neutralization titer. Five samples had evidence of IgM against RVFV, none of which had evidence of IgG. Of the 3 samples tested for neutralizing antibodies, only 1 neutralized RVFV (data not shown). No other bunyaviruses were available for comparison.

**Table 3:** Results of Immunologic assays for serum samples that tested IgM positive only for alphaviruses, Lassa Diagnostic Laboratory, Kenema, Sierra Leone, October 2006–October 2008*

Sample no.	CHIKV ELISA			Alphavirus PRNT			
	IgM	IgG		CHIKV	ONNV	SFV	SINV
051–5	0.34	0.07		160	2,560	10	10
055–1	0.34	0.00		160	2,560	<10	10
132–1	1.32	0.00		640	2,560	40	<10
168–1	0.76	0.03		10	640	10	10

*Patient samples were tested for IgM and IgG reactivity in a CHIKV ELISA. Samples with only IgM were tested for their ability to neutralize specific alphaviruses, CHIKV, chikungunya virus; PRNT, plaque-reduction neutralization test; ONNV, o'nyong-nyong virus; SFV, Semliki Forest virus; SINV, Sindbis virus.

Comparative PRNTs for the ebolaviruses used EBOV, SUDV, and TAFV, all of which are known to have circulated in Africa. Eighteen samples were IgM positive only by ELISA. Of these, 14 had sufficient volume for PRNT against all 3 ebolaviruses (Table 4). Eight of these neutralized EBOV, 7 of which were 4 times more reactive to EBOV than to the other ebolaviruses tested. One sample was 4 times more reactive to SUDV, and 1 neutralized TAFV but only at a 1:10 dilution. Four patient samples did not neutralize any of the ebolaviruses tested. We found no correlation between ELISA OD and neutralization titer. We did not test samples with evidence of MBGV-specific IgM by PRNT because no known neutralizing antibody was available to use as a control.

**Table 4:** Results of immunologic assays for serum samples testing IgM positive only for ebolaviruses, Lassa Diagnostic Laboratory, Kenema, Sierra Leone, October 2006–October 2008*

Sample no.	ELISA			PRNT		
	IgM	IgG		EBOV	SUDV	TAFV
060–1	0.35	0.00		40	<10	<10
076–1	0.45	0.00		40	<10	10
085–1	0.20	0.00		40	<10	<10
090–1	0.26	0.06		40	<10	<10
118–2	0.23	0.00		40	<10	<10
119–1	0.24	0.06		<10	<10	<10
120–1	0.38	0.00		<10	<10	10
121–1	0.58	0.00		<10	<10	<10
122–1	0.66	0.00		40	<10	<10
125–1	0.24	0.00		ND	ND	ND
129–2	0.37	0.00		<10	<10	<10
130–1	0.40	0.03		<10	40	<10
131–1	0.25	0.00		<10	<10	<10
132–1	0.21	0.09		ND	ND	ND
143–1	0.30	0.00		ND	ND	ND
144–1	0.35	0.06		40	<10	<10
182–1	0.38	0.00		ND	ND	ND
261–1	0.29	0.00		10	<10	<10

*Patient samples were tested for IgM and IgG reactivity in an EBOV ELISA. Samples with IgM only were tested for their ability to neutralize specific ebolaviruses, PRNT, plaque-reduction neutralization test; EBOV, Ebola virus; SUDV, Sudan Gulu strain; TAFV, Taï Forest (Cȏte d’Ivoire) viruses.

## Discussion

In West Africa, as in many regions of Africa, infectious disease is part of everyday life. The cause of disease is often unknown or incompletely understood because of nonspecific clinical features, lack of diagnostic laboratory support, or little or no knowledge about disease prevalence in a region (*23*). Within the LASV-hyperendemic region, Lassa fever is always possible, but early signs and symptoms are similar to those of other viral, bacterial, and rickettsial diseases, which can confound a clinical diagnosis (*24*). Our aim was to investigate other viral diseases that cause acute febrile illnesses originally thought be Lassa fever. We investigated arthropod-borne and hemorrhagic fever viruses that were likely to occur in the region. More than 25% of the LASV-negative patients had evidence of infection with other arthropod-borne or hemorrhagic fever viruses.

Using only retrospective field-collected samples limited the analysis and thus our conclusions. In a prospective study, patients would be sampled during the acute phase and again during the convalescent phase of illness. Virus isolations, antigen-detection ELISAs, and/or reverse transcription PCR would be attempted on all acute-phase samples. Testing acute- and convalescent-phase serum would enable both IgM and IgG testing and confirm positive results by a >4-fold increase in neutralizing titer. In this retrospective study we had only acute-phase samples; therefore, our results can be considered presumptive only.

Because the samples submitted to the Lassa Diagnostic Laboratory were from patients with acute illness, IgM-capture ELISAs were used to detect the earliest antibody elicited in response to viral infection. We found evidence of IgM for flaviviruses (DENV, WNV, and YFV); the bunyavirus RVFV; the alphavirus CHIKV; and the filoviruses EBOV and MBGV (Table 2). We tested IgM-positive samples for IgG when possible. Most samples exhibited only IgM or very low IgG levels, suggesting acute-phase disease or the beginning of class switching (data not shown). Exceptions were the samples that had CHIKV antibodies; 6 of the 10 patients had higher IgG than IgM against CHIKV, suggesting late acute-phase or early convalescent-phase infection.

PRNT is the laboratory standard for immunologic assays. It measures in vitro virus neutralization and is the most virus-specific serologic test to confirm immunologic test results. Testing CHIKV-positive serum demonstrated that the patients were more likely to have been infected by ONNV, a related but separate virus species. The viruses can be distinguished genetically by sequence analysis but with greater difficulty by serologic testing (*25*). Antibodies to the 2 viruses are generally distinguishable only by PRNT. We demonstrated that the CHIKV ELISA we used can detect antibodies to both viruses and confirmed the results by PRNT (Table 3). Most patient samples that reacted in the CHIKV IgM ELISA were ONNV upon confirmatory testing in the PRNT (Table 3). PRNT results for other viruses provided some additional information but at times were incomplete because of limitations of available virus strains or appropriate positive controls. We found clear evidence for YFV infections in the samples tested, but data were incomplete for other flaviviruses. PRNT results for RVFV and MBGV infections were similarly inconclusive. PRNT results for the ebolaviruses clearly indicated that most resulted from EBOV infections (Table 4). We found evidence that SUDV was responsible for 1 infection but no evidence for TAFV infection, the only ebolavirus isolated in West Africa. In the ebolavirus PRNTs, we did not include the newest discovered ebolavirus, Bundibugyo virus, which cross-reacts with EBOV in immunoassays (*26*). Ebolavirus infections in Sierra Leone might be the result of Bundibugyo virus or an ebolavirus genetic variant and not EBOV.

Several arthropod-borne viruses are known to circulate in West Africa (*23*). Using ELISA to look for IgM and IgG, we found indication of infections with the flaviviruses DENV, WNV, and YFV; the bunyavirus RVFV; the alphavirus CHIKV (shown to be ONNV by PRNT); and the filoviruses EBOV and MBGV. Evidence of flavivirus infections was not unexpected. DENV, WNV, and YFV infections have been reported in Sierra Leone and the surrounding region (*23*,*27*–*30*). CHIKV is thought to be enzootic in West Africa, maintained in a sylvatic cycle involving nonhuman primates and *Aedes* species mosquitoes (*25*). ONNV is a distinct virus species but closely related to CHIKV. CHIKV fever is described throughout the region, but ONNV disease has not been described in this immediate region. In 2003, an outbreak of ONNV was reported in Cȏte d’Ivoire (*31*).

RVFV, a bunyavirus in the *Phlebovirus* genus, is endemic to East and South Africa, but may not be established in West Africa (*32*–*34*). In this study, we found evidence of RVFV IgM and confirmation of at least 1 of them as neutralizing the virus. CCHFV, another bunyavirus in the *Nairovirus* genus, circulates in West Africa, but we found no evidence of CCHFV infections in any patient samples tested (*35*). The filoviruses represented the largest group of patient samples that reacted in our study. This finding was surprising because no filovirus has been reported in the region or in West Africa other than the initial isolation of TAFV in Cȏte d’Ivoire (*36*). These serologic results provide evidence that ebolaviruses are circulating and infecting humans in West Africa. All of the ebolavirus-reactive samples demonstrated only IgM and no evidence of IgG, suggesting acute infection. PRNT results indicated that the infecting virus was most closely related to EBOV, except for 1 SUDV-reactive patient sample. This finding was unexpected because our assumption was that any ebolavirus would more likely be TAFV, the only species described in West Africa. Although the serum samples were able to neutralize EBOV only at a low level (1:40 dilution), it is possible that the virus is an EBOV genetic variant. This presumptive diagnosis of EBOV infection extends the ebolavirus geographic region to Sierra Leone and the surrounding region. The MBGV-reactive samples, similar to the ebolavirus samples, had evidence only of IgM, suggesting acute infection. Unfortunately, we were unable to determine whether the samples could neutralize any MBGV because we were unable to acquire a known neutralizing serum to use as a positive control.

Our presumptive results provide some insight into the other viruses causing acute disease in the patients whose samples were submitted to the Lassa Diagnostic Laboratory. Although our results are not definitive, they demonstrate arthropod-borne and hemorrhagic fever viruses that should be considered when Lassa fever is suspected. These continued studies will add to the body of knowledge for Lassa fever and other arthropod-borne diseases and hemorrhagic fevers that occur naturally within Sierra Leone and West Africa.
